# Biosynthesis of silver nanoparticles from macroalgae *Hormophysa triquetra* and investigation of its antibacterial activity and mechanism against pathogenic bacteria

**DOI:** 10.1038/s41598-024-84760-y

**Published:** 2025-01-20

**Authors:** Shazia Bibi, Mohammed H. Abu-Dieyeh, Mohammad A. Al-Ghouti

**Affiliations:** 1 Department of Biological and Environmental Sciences, College of Arts and Sciences, Qatar University, P. O. Box: 2713, Doha, Qatar; 2 Biological Science Program, Department of Biological and Environmental Sciences, College of Arts and Sciences, Qatar University, P. O. Box: 2713, Doha, Qatar; 3 Environmental Science Program, Department of Biological and Environmental Sciences, College of Arts and Sciences, Qatar University, P. O. Box: 2713, Doha, Qatar

**Keywords:** Macroalgae, *Hormophysa triquetra*, Silver nanoparticles, Green synthesis, Antibacterial activity, Mechanism of action, Plant biotechnology, Environmental sciences, Nanoscience and technology

## Abstract

In this study, brown macroalgae *Hormophysta triquetra* (HT) collected from the Qatari coast is used to biosynthesize silver nanoparticles (AgNPs) from its aqueous (AQ), chloroform: methanol (MCF), and ethanolic extracts (ET). The NPs are characterized using Transmission electron microscopy (TEM), Fourier transform infrared spectroscopy (FTIR), X-ray diffraction (XRD), Gas chromatography/Mass spectrometry (GC/MS) and X-ray photoelectron spectroscopy (XPS). The NPs were evaluated for their antibacterial activities by disc-diffusion method and their minimum inhibitory concentrations (MIC) were assessed. The NPs synthesized through biological process exhibited significant antibacterial efficacy against *Escherichia coli*,* Bacillus subtilis*,* Staphylococcus aureus*,* Pseudomonas stutzeri*, and *Pseudomonas fragi* for all the three NPs. AQ-AgNP and ET-AgNP showed higher zones of inhibition for *P. fragi* with inhibitory zones of 22.5 mm and 25 mm respectively. On the other hand, MCF-AgNP showed a higher zone of inhibition for *E. coli* with an inhibition zone of 23.5 mm. The NPs inhibited the growth of bacterial strains by deforming their structure and forming pits. The results revealed that macroalgae HT could be used as a potential candidate to produce AgNPs and have efficient antibacterial activities against both types of bacteria i.e., Gram-positive (*B. subtilis and S. aureus*) and Gram-negative (*E. coli*,* P. stutzeri*, and *P. fragi*).

## Introduction

Recently, the use of NPs has tremendously increased for different purposes. NPs are in demand due to their exclusive shapes, sizes, and a larger surface area to volume. The size of NPs varies from 1 μm to 100 nm^1^. Metallic NPs are significantly studied due to their characteristic features such as large surface area, electrical, chemical, catalytic, and optical properties^2,3^. Generally, NPs are synthesized by physiochemical, electrochemical, photochemical, and heat evaporation methods that have limitations and disadvantages. These methods to synthesize NPs are costly and utilize hazardous chemicals that are toxic and have potential to harm environmental and biological entities^2,4^. Because of their harmful properties, the use of such NPs is restricted in biomedical field. In this context, Green nanotechnology can be regarded as an environmentally sustainable approach in which NPs are synthesized from living organisms such as plants, fungi, and bacteria^5^. This process is efficient both environmentally and economically^6^. Different types of NPs exist such as AgNPs, gold nanoparticles (AuNPs), copper nanoparticles (CuNPs), and platinum nanoparticles (PtNPs)^7,8^. These biologically prepared metallic NPs are reported to alter the biological activities of different bacteria^9^ and hence, it could potentially serve as a candidate for treating bacterial infections/diseases. Recently, the biosynthesis of AgNPs has surged. The biosynthesis of AgNPs utilizes a specific concentration of metal salt silver nitrate (AgNO_3_). Through the bio reduction of Ag, AgNPs are biosynthesized. Ag has been utilized as a safe inorganic antimicrobial mediator with the ability to inhibit or kill 650 types of pathogenic microbes^10^. Studies demonstrate that AgNPs behave as strong inhibitors and develop a protective barrier against Gram-positive and Gram-negative bacteria^11^. *E. coli*,* Salmonella enteritidis*,* S. aureus*,* P. aeruginosa*,* Candida albicans*,* Acinetobacter baumannii*,* Shigella sonnei*,* Micrococcus luteus*,* Streptococcus pyogenes*,* Klebsiella pneumoniae*, and *Proteus vulgaris* are some of the species that are inhibited by AgNPs^12–16^. These inhibitory effects are achieved through neutralization of surface charge which leads to variation in the membrane permeability of bacteria leading to bacterial death^17^. Further, AgNPs role in production of reactive oxygen species (ROS) that damage defense system of the cell, are responsible for anti-oxidation processes causing mechanical breaking of the membrane^18^. Moreover, AgNPs can pass through the bacterial biofilms and inhibit its development through gene suppression^19^. In aqueous medium, metallic ions from the NPs are released and absorbed through the microbial cell membranes. Consequently, the Ag^+^ ions interact with the protein functional groups like thiol leading to varying structural and enzymatic activities resulting in abnormal physiological events^20^.

Approximately 70% of the surface of Earth is oceans that have incredible biodiversity. Marine species, with their wide variety, offers exclusive advantages. Among the many available marine species, macroalgae is a crucial element of the marine ecosystem, which is considered potent for the presence of valuable secondary and biologically active compounds^21^. Seaweeds/macroalgae use sunlight, carbon dioxide (CO_2_) and water (H_2_O) to synthesize carbohydrates^22^. Macroalgae occur in marine benthic habitats and are multicellular entities found either free-floating or bound with rocks in the marine environment^23,24^. They are composed of different compounds such as carbohydrates, proteins, lipids, minerals (ash), macro-minerals (Na, K, Ca, Mg, and P), and trace elements (Fe, Zn, Cu, Mn, and I)^25^. Macroalgae are home to significant sources of polyphenols, proteins, minerals, iodine, and vitamins. Through secondary metabolites production and adaptive mechanisms, macroalgae can survive in harsh environmental conditions that include extreme temperatures and pH, salinity and other such factors^26^. Different seaweeds are used to biosynthesize NPs to be utilized as antimicrobial agents. For instance, the aqueous extract of macroalgae *Turbinaria conoides* has been used to synthesize AgNP of 96 nm size that has shown high toxicity towards pathogenic bacterial species such as *B. subtilis* and *K. planticola*^27^. Comparatively, the methanolic extract of *Sargassum polycystum* is used to biosynthesize AgNPs of a relatively smaller size 5–7 nm. These NPs also showed inhibitory action towards human pathogens^28^. The sizes and shapes of NPs depend on the concentration and type of precursor used. AgNPs are mostly reported to be spherical in shape whereas other NPs where a different precursor and conditions are used can have different shapes like spherical, rectangular, triangular, and radial. Such NPs are biosynthesized from all three types of seaweed: green (*Caulerpa pelteta*), red (*Hypnea Valentiae*), and brown (*S. mariocystum*). Other macroalgae used to biosynthesize NPs include *S. longifolium*,* Ulva lactuca*,* Corallina officinalis*,* Gracilaria corticate*, and *H. musciformis*^29^.

In this research, HT from Qatari coast was utilized to synthesize AgNPs from the AQ, ET, and MCF extracts. To evaluate the characteristics of biosynthesized AgNPs, various spectroscopic techniques that include UV-VIS spectroscopy, TEM, FTIR and others will be used. The mechanism with which NPs work against bacterial strains will also be examined through SEM analysis. To our knowledge, this study is the first study in Qatar where macroalgal AgNPs are biologically synthesized and tested for their antibacterial potential. The findings of this research may serve as a foundation of future studies examining the composition and biological characteristics of macroalgae especially in this region.

## Results

### Biosynthesis of AgNPs and confirmation by UV-VIS spectroscopy

**Fig. 1:**
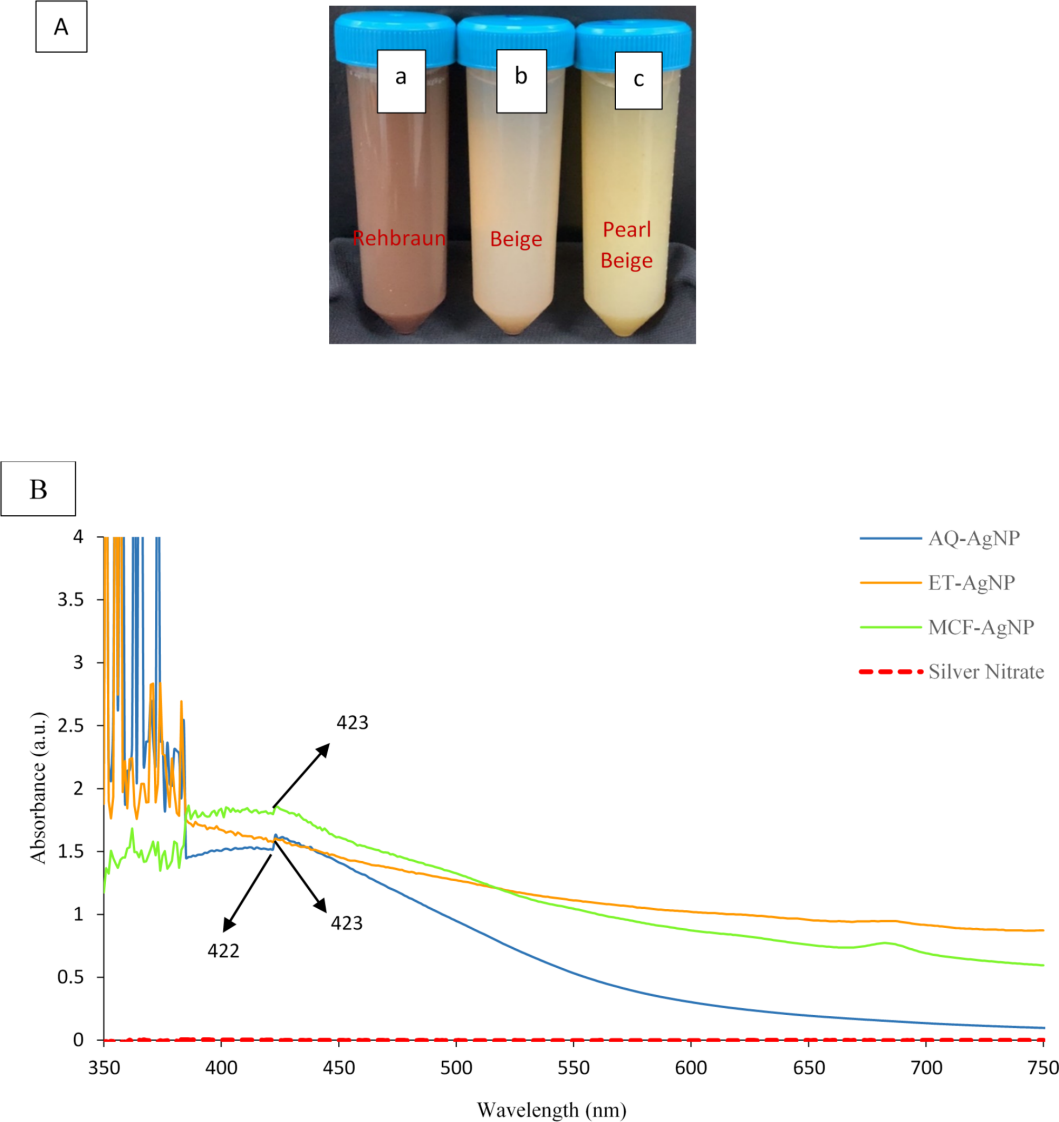
(**A)** Post-incubation color changes from colorless to: (**a**) Rehbraun for Aqueous Silver Nanoparticle (AQ-AgNP), (**b**) Beige for Ethanolic Silver Nanoparticle (ET-AgNP), and (**c**) Pearl Beige for Methanol-Chloroform Silver Nanoparticle (MCF-AgNP), (**B**) UV-VIS spectra analysis of AgNPs synthesized from aqueous (AQ), ethanolic (ET), methanol-chloroform (MCF) extracts, and the control AgNO_3_.

The process of AgNP formation starts when the silver source interacts with the functional groups in the extract. This reaction causes the reduction of silver, resulting in the creation of AgNPs. The first indication of biosynthesis is the color change of the initially colorless solution to a rehbraun color for AQ-AgNP (Fig. 1. A(a)). In contrast, when the colorless AgNO_3_ interacts with ET and MCF extract powders, it turns cloudy, with the final colors being beige for ET-AgNP and pearl-beige for MCF-AgNP (Fig. 1. A(b, c)). After visual confirmation, secondary confirmation was done by UV-VIS spectrophotometer based on the surface plasmon resonance (SPR) bands. For the three biosynthesized NPs, AQ-AgNP, ET-AgNP, and MCF-AgNP, the bands appeared at 422 nm, 423 nm, and 423 nm correspondingly as shown in Fig. 1B.

The pH of the biosynthesized AQ-AgNP, ET-AgNP, and MCF-AgNP were 6.5, 7.29, and 6.22 respectively. The working pH at which nanoparticles are synthesized play an important role in its stability and the reduction process of Ag. Moreover, it also assists in the antibacterial activity of the biosynthesized AgNPs^30^.

### TEM analysis

**Fig. 2:**
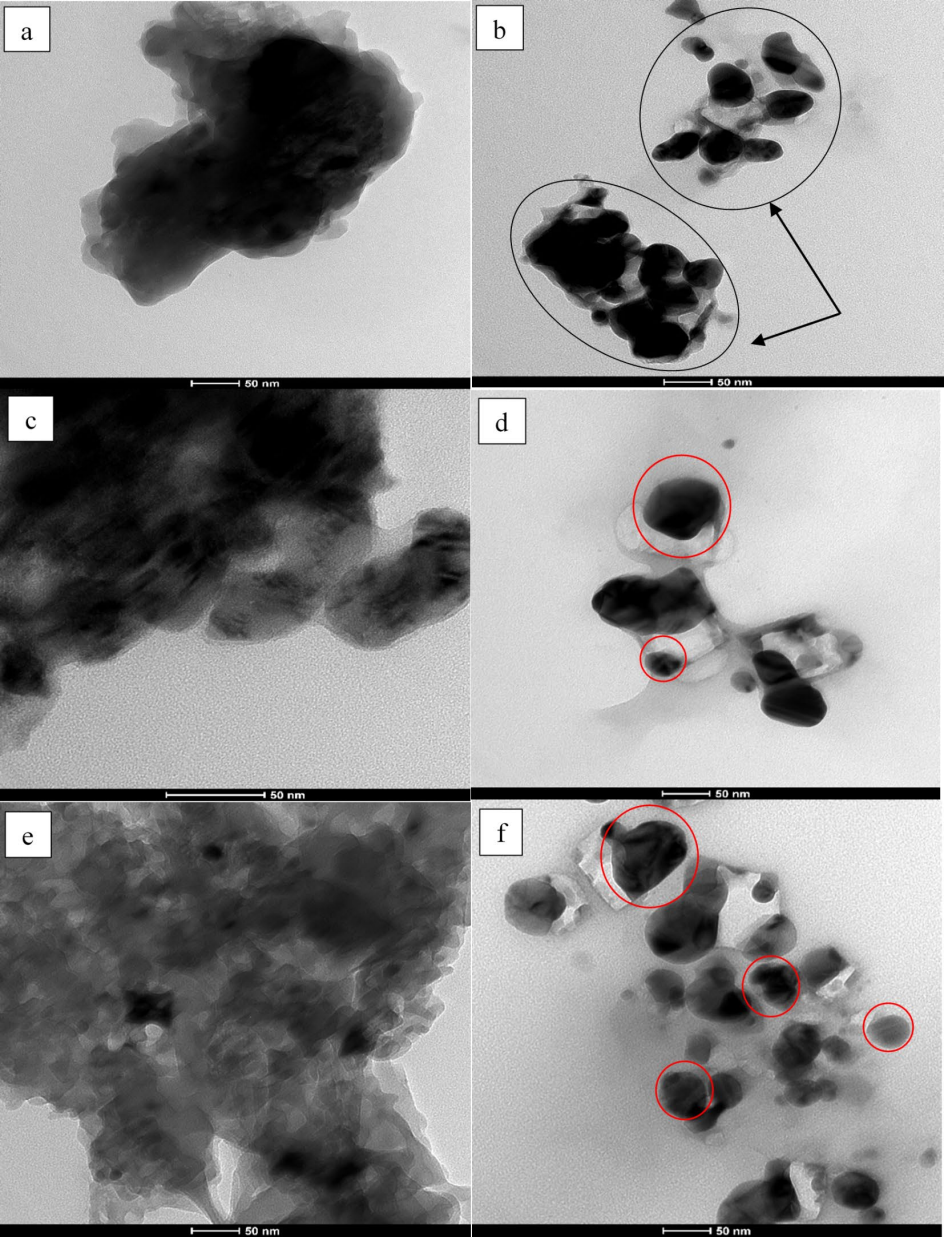
TEM images of (**a**) Aqueous extract (**b**) Aqueous Silver Nanoparticle (**c**) Ethanolic extract (**d**) Ethanolic Silver Nanoparticle (**e**) Methanol-chloroform extract, and (**f**) Methanol-Chloroform Silver Nanoparticle. Arrowed black circles indicate agglomeration of AgNPs. Red circles indicate single crystal of AgNP.

TEM analysis of the extracts is shown in Fig. 2a, c and e while Fig. 2b, d and f shows the biosynthesized AgNPs. TEM analysis revealed that the AgNPs synthesized from the three types of extracts were spherical in shape with sizes varying between 10 nm and 70 nm. The image (Fig. 2b circled) indicates possible agglomeration which could be attributed to the capping process of biosynthesis of AgNPs^31^ while circled red in Fig. 2d and f indicates the crystalline structure and different sizes of NPs. TEM of extracts were done as a control that clearly shows irregular structure of extract used with no crystalline structures before reacting with AgNO_3_.

### FTIR analysis

**Fig. 3:**
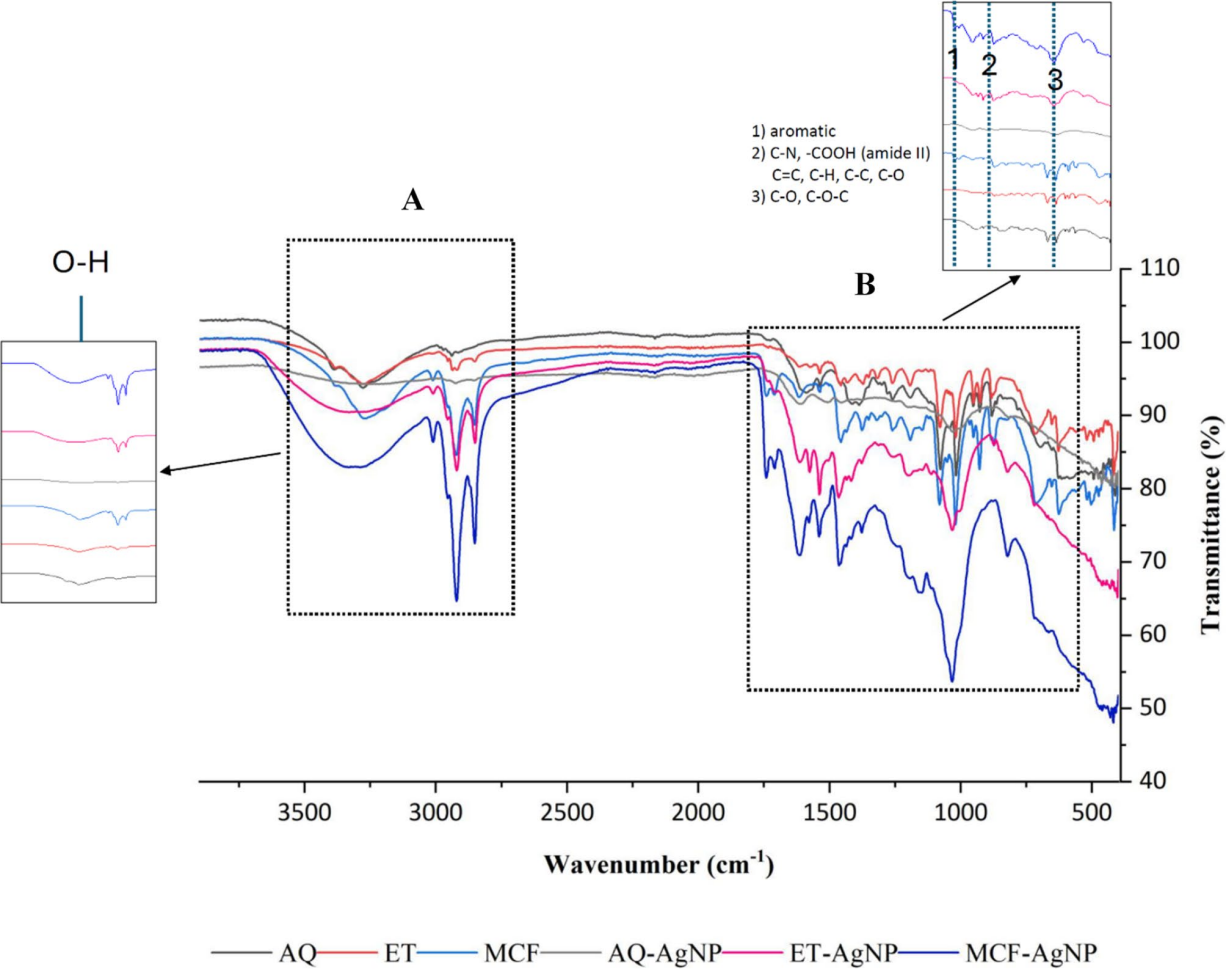
FTIR spectra of extracts: Aqueous (black), ethanolic (red), methanol: chloroform (blue) and silver nanoparticles: Aqueous Silver Nanoparticle (grey), ethanolic Silver Nanoparticle (pink), and methanol-chloroform Silver Nanoparticle (dark blue). Regions A and B show peaks for different functional groups.

FTIR spectra of the extracts and the biosynthesized NPs are shown in Fig. 3. The differences between the peaks of crude extracts and their respective NPs indicate the involvement of those functional groups in their synthesis. Further, intensification of certain peaks in the spectra of NPs demonstrates the role of those groups in capping and stabilizing the formed AgNPs. The spectra reveal the presence of broadbands between 3000 cm^− 1^ to 3500 cm^− 1^ in both ET and MCF extracts where as in AQ extract, the region is flattened (Fig. 3, region A). On the right side of the spectra, another region B is shown with smaller peaks between 550 cm^− 1^ to 1700 cm^− 1^. These peaks correspond to aromatic functional groups, C-N, COOH, C-C, C-O, and C-O-C groups. In comparison, AQ-AgNP shows peaks at 3262.63 cm^− 1^, 1032.46 cm^− 1^, 462.87 cm^− 1^, and 418.46 cm^− 1^, 403.68 cm^− 1^ corresponding to O-H stretching, C-O stretching of secondary alcohols^32^, cycloalkane^33^, and alkyl halides (C-X)^34^. The FTIR spectra of ET-AgNP shows peaks at 3331.68 cm^− 1^, 2920.38 cm^− 1^, 2850.72 cm^− 1^, 1614.65 cm^− 1^, 1575.93 cm^− 1^, 1538.85 cm^− 1^, 1465.06 cm^− 1^, 1032.26 cm^− 1^, and 403.59 cm^− 1^ attributed to O-H stretch^35^, C-H stretching of -CH_2_^36,37^, skeletal vibration of C = C^38^, symmetric stretching vibration of -COOH groups from amide II^39^, C = C stretching^40^, C-H bending^41^, C-N stretching^42^, C-O stretching, and alkyl halide (C-X)^34^. Lastly, MCF-AgNP show peaks at 3289.82 cm^− 1^, 3010.79 cm^− 1^, 2920.99 cm^− 1^, 2851.69 cm^− 1^, 1741.26 cm^− 1^, 1612.71 cm^− 1^, 1540.25 cm^− 1^, 1464.31 cm^− 1^, 1377.61 cm^− 1^, 1148.22 cm^− 1^, 1033.21 cm^− 1^, 822.06 cm^− 1^, 430.78 cm^− 1^, and 419.23 cm^− 1^ associated with O-H stretching^43^, CH = CH asymmetric stretching^44^, C-H stretching^45^, C-H symmetric stretching^46^, C = O stretching from ester group^47^, aromatic stretching vibrations^48^, C-C stretching^49^, C-N stretching^50^, COO- antisymmetric stretching^51^, C-O groups^52^, C-O-C stretching^53^, C-O-C asymmetric bending^54^, aromatic rings^55^, and alkyl halides^34^.

### XRD analysis

**Fig. 4:**
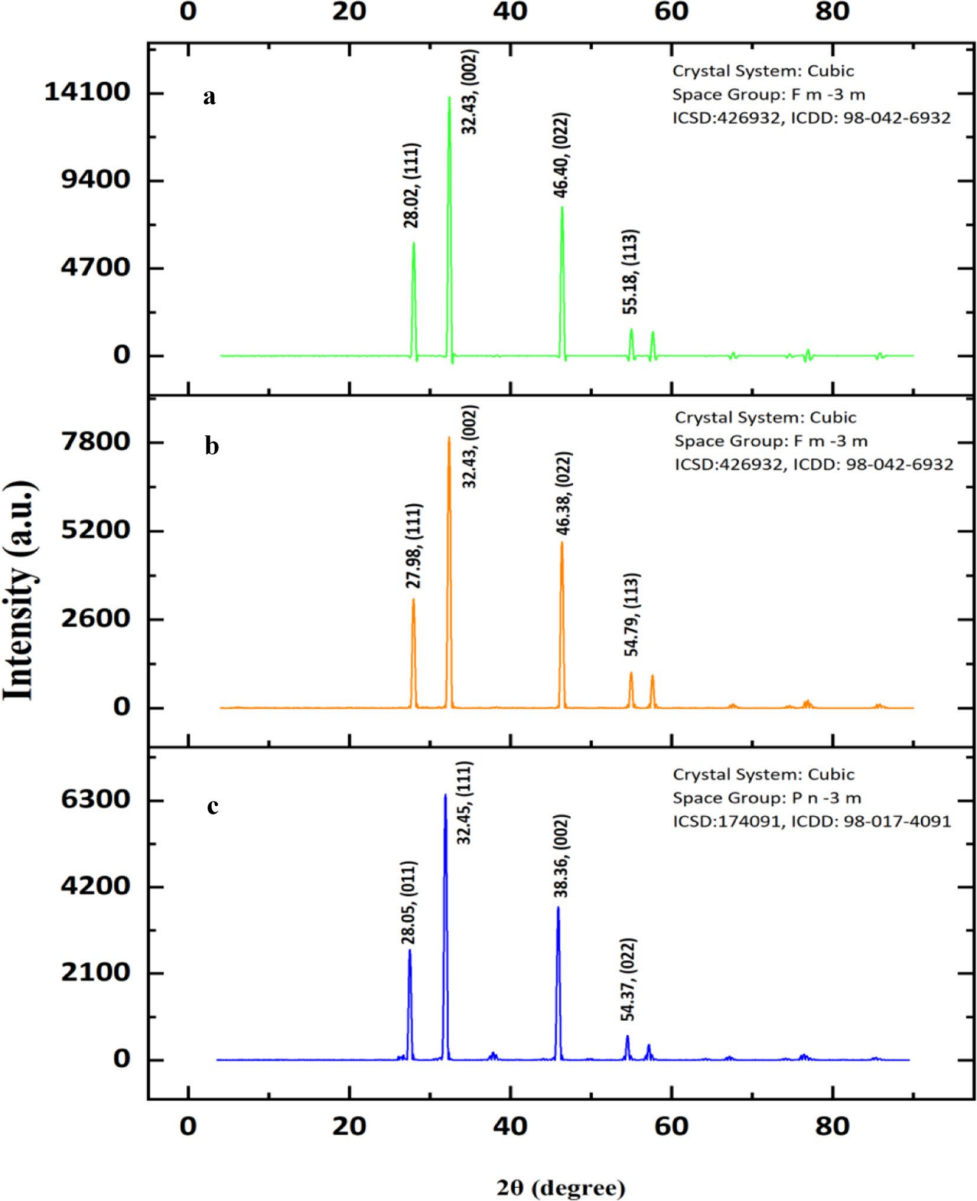
XRD patterns of biosynthesized AgNPs, (**a**) Methanol: Chloroform Silver Nanoparticles, (**b**) Ethanolic Nanoparticles, and (**c**) Aqueous Silver Nanoparticles.

The XRD pattern of all the AgNPs are shown in Fig. 4. The diffraction peaks of AQ-AgNP at 2θ values are 28.05°, 32.45°, 38.36°, and 54.37° attributed to (011), (111), (002), and (022) (Fig. 4c) (Ref: International Center for Diffraction Data (ICDD: 98-017-4091)). ET-AgNPs indicate diffraction peaks at 2θ values of 27.98°, 32.43°, 46.38°, and 54.79°with (111), (002), (022), and (113) (Fig. 4b) (Ref: ICDD: 98-042-6932). Lastly, MCF based AgNP showed diffraction peaks at 2θ values of 28.02°, 32.43°, 46.40°, and 55.18° with (111), (002), (022), and (113) as shown in Fig. 4a (Ref: ICDD: 98-042-6932). The crystallite size of the biosynthesized AgNPs are 41.5 nm, 34.1 nm, and 59.5 nm for AQ-AgNP, ET-AgNP, and MCF-AgNP. The full width at half maximum (FWHM) values obtained for 2θ values of 32.45°, 32.43°, and 32.43° are 0.1791, 0.2175, and 0.1248 respectively. Moreover, the wavelength ($\:\varvec{\lambda\:}$) of x-ray used is 0.15.

### GC/MS analysis

The GC/MS chromatograms showed different peaks for the NPs revealing various compounds listed in Table 1. These compounds were obtained after comparing the retention time of the peaks with those listed in the National Institute of Standards and Technology (NIST) library. Nanoparticles synthesized from AQ extract of macroalgae showed 5 peaks on the GC/MS chromatogram. The ET-AgNP GC/MS chromatogram recorded a total of 6 peaks whereas 8 peaks were found in the chromatogram of MCF-AgNP. Overall, 19 compounds were identified from the chromatograms of all the three NPs with potential antibacterial role.

**Table 1:** Compounds name, Retention time (min), scoring index (SI), chemical formula and molecular weight (g/mol) of the compounds found in the GC/MS spectra of Aqueous Silver Nanoparticle, Ethanolic Silver Nanoparticle, and methanol-chloroform silver nanoparticle.

Compound Name	Retention time (min)	SI	Chemical formula	Molecular weight (g/mol)
Aqueous silver nanoparticle				
Octanoic acid, 3-phenylprop-2-enyl ester	1.045	35	C_17_H_24_O_2_	260
Hexadecenoic acid, 2-hydroxy-1-(hydroxymethyl) ethyl ester	12.975	69	C_19_H_38_O_4_	330
Octadecanoic acid, 2-3-dihydroxypropyl ester	14.1	71	C_21_H_42_O_4_	358
6,10,14,18,22-Tetracosapentaen-2-ol, 3-bromo-2,6,10,15,19,23-hexamethyl-	14.75	92	C_30_H_51_BrO	506
Pregna-5, 16-dien-20-one, 3-hydroxy-, (3β)	20.47	78	C_21_H_30_O_2_	314
Ethanolic silver nanoparticle				
Ethane, 1, 1, 2-trichloro	1.17	56	C_2_H_3_Cl_3_	132
Dodecanal lauraldehyde	10.145	87	C_12_H_24_O	184
Octadecanoic acid, ethyl ester	11.19	82	C_20_H_40_O_2_	312
9-Octadecynoic acid, methyl ester	12.165	85	C_19_H_34_O_2_	294
Triacontyl acetate	14.84	60	C_32_H_64_O_2_	480
Campesterol	20.5	60	C_28_H_48_O	400
Methanol-chloroform silver nanoparticle				
Ethane, 1,1,2-trichloro	1.17	57	C_2_H_3_Cl_3_	132
Octadecanoic acid, 3-hydroxy-2-tetradecyl-, methyl ester	9.5	71	C_33_H_66_O_3_	510
6,9-Octadecadienoic acid, methyl ester	10.59	73	C_19_H_34_O_2_	294
Aromandendrene	11.575	71	C_15_H_24_	204
Triacontanoic acid, methyl ester	13.06	83	C_31_H_62_O_2_	466
7-Octene-2,4-dione	14.82	52	C_8_H_12_O_2_	140
Cholesterol	17.655	87	C_27_H_46_O	386
Campesterol	20.49	66	C_28_H_48_O	400

### XPS analysis

**Fig. 5:**
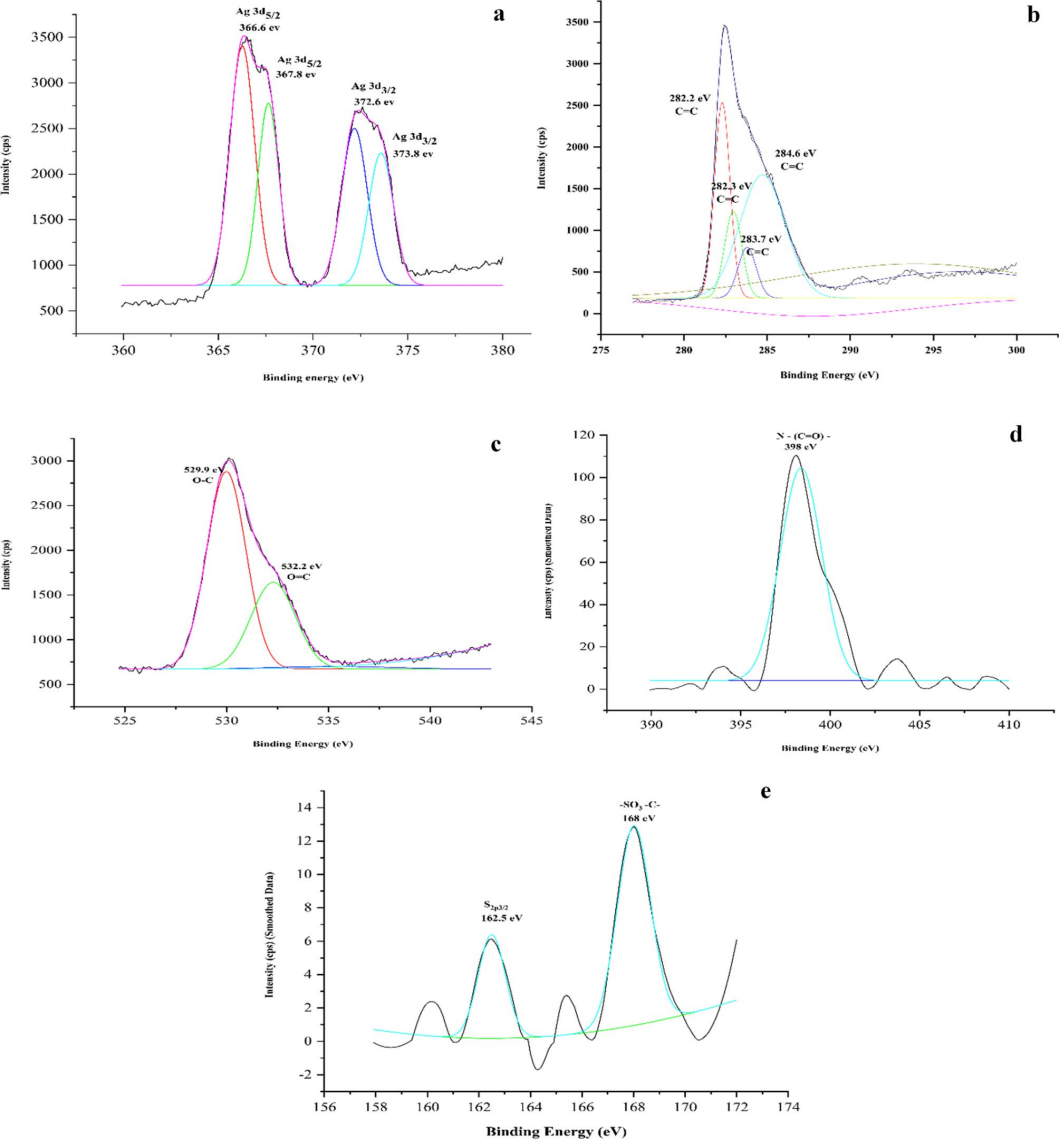
XPS graphs of Aqueous Silver Nanoparticles showing elements (**a**) Silver (**b**) Carbon (**c**) Oxygen (**d**) Nitrogen, and (**e**) Sulphur.

The XPS analysis demonstrated the composition, and oxidation states of the elements present in the biosynthesized AgNPs as shown in Fig. 5. Silver, carbon (C), oxygen (O), nitrogen (N) and sulfur (S) were analyzed by XPS analysis. In AQ-AgNP, Ag metal appeared at peaks adjacent to each other with binding energies of 366.6 eV, 367.8 eV, 372.6 eV, and 373.8 eV. The first two peaks correspond to Ag 3d_5/2_ while the other two peaks correspond to Ag 3d_3/2_. The element C shows a single peak, however, when deconvoluted, it showed four peaks close to each other at 282.2 eV, 282.3 eV, 283.7 eV, and 284.6 eV. All the peaks showed C1s attributed to the sp2 carbon configuration of C = C^56^. Oxygen exists in O1s which upon deconvolution showed two peaks for chemical states of O-C at 529.9 eV and O = C at 532.2 eV^57^. The N1s showed a peak at 398 eV which corresponds to a chemical state of -N-(C = O)-^58^. Moreover, for element S, two peaks were observed: S 2p_3/2_ at 162.5 eV and -SO_3_-C- at 168 eV^59^.

**Fig. 6:**
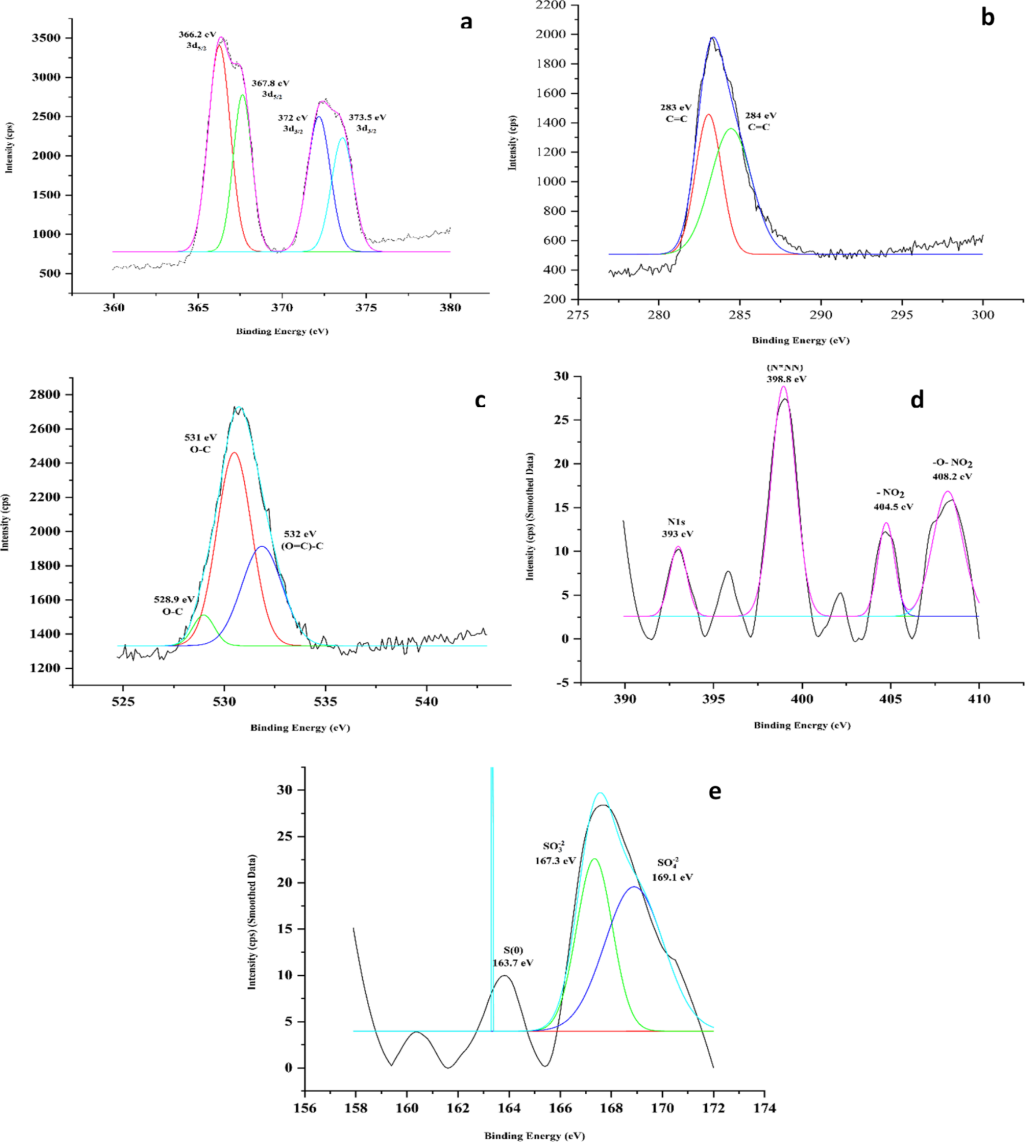
XPS graphs of Ethanolic Silver Nanoparticles showing elements (**a**) Silver (**b**) Carbon (**c**) Oxygen (**d**) Nitrogen and (**e**) Sulphur.

Looking at the XPS analysis of ET-AgNP (Fig. 6), the same elements were analyzed. For Ag, deconvolution showed 4 peaks at 366.2 eV, 367.8 eV, 372 eV, and 373.5 eV respectively. The first two peaks correspond to 3 d_5/2_ while the rest to 3 d_3/2_. The C1s demonstrated two peaks at 283 eV and 284 eV relating to chemical state of C = C^60^. The O1s spectra showed three peaks at 528.9 eV, 531 eV, and 532 eV of which two corresponds to chemical state of O-C, and the last relates to (O = C)-C^61^. N1s showed peaks at 393 eV, 398.8 eV, 404.5 eV, and 408.2 eV corresponding to chemical states of elemental N, azide (N*NN), nitro (-NO_2_), and nitrooxy (-O-NO_2_)^62,63^. XPS spectra for elemental S showed peaks at 163.7 eV, SO_3_ ^− 2^ at 167.3 eV, and SO_4_ ^− 2^ at 169.1 eV^59^.

**Fig. 7:**
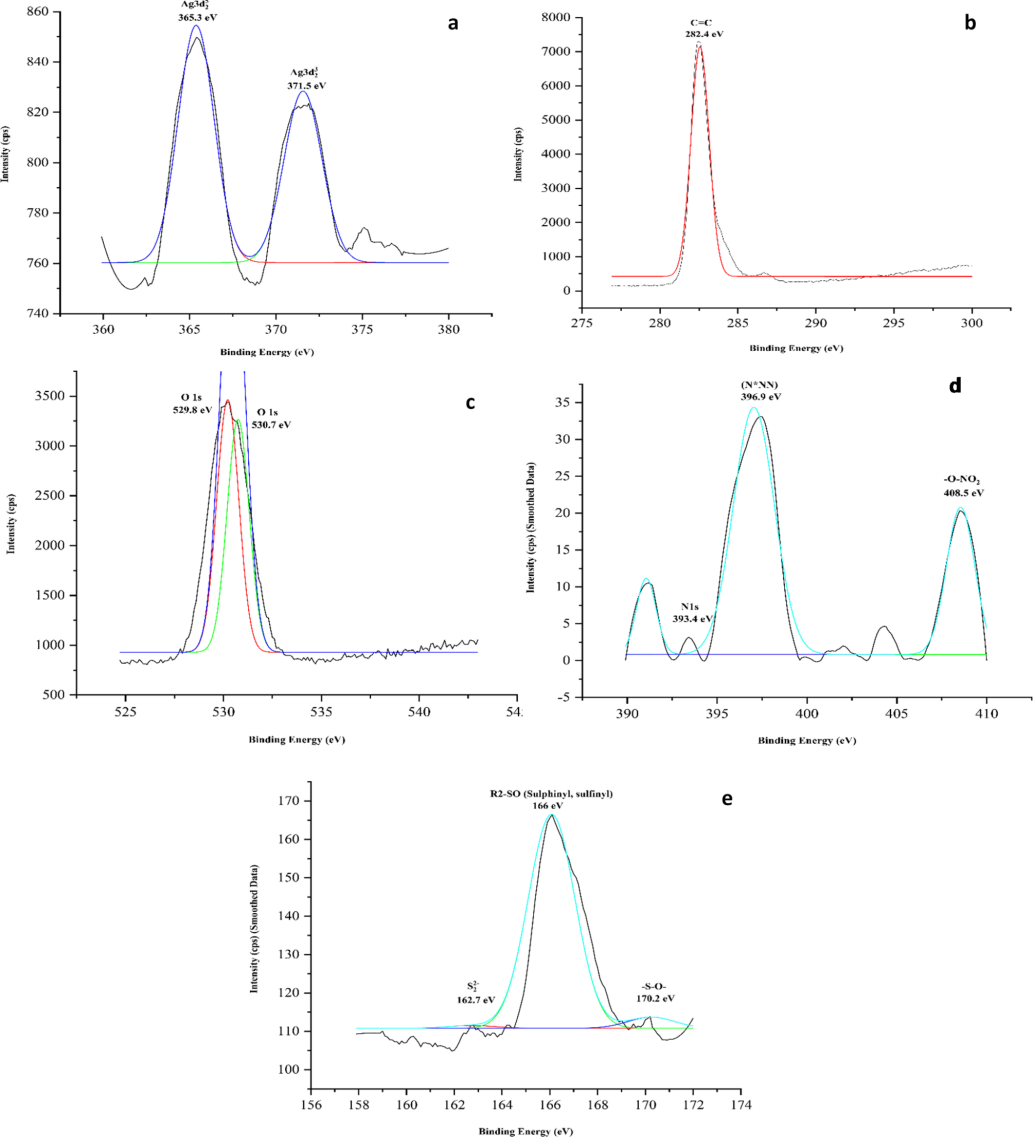
XPS graphs of Methanol-chloroform Silver Nanoparticle showing elements (**a**) Silver (**b**) Carbon (**c**) Oxygen (**d**) Nitrogen and (**e**) Sulphur.

XPS analysis of MCF-AgNP (Fig. 7) showed two distinct peaks for Ag at 365.3 eV and 371.5 eV both with chemical state of Ag 3d_3/2_. C1s spectra showed a single peak for C = C at 282.4 eV whereas deconvoluted O1s spectra demonstrated two peaks close to each other at 529.8 eV and 530.7 eV respectively. N1s deconvoluted spectra showed peaks at 393.4 eV for elemental N, 396.9 eV for N*NN, and 408.5 eV for -O-NO_2_ correspondingly^63^. Deconvoluted spectra of S showed peaks at 162.7 eV for S_2_ ^− 2^, 166 eV for sulphinyl (R2-SO), and 170.2 eV for -S-O-^63,64^.

### Antibacterial activity

**Fig. 8:**
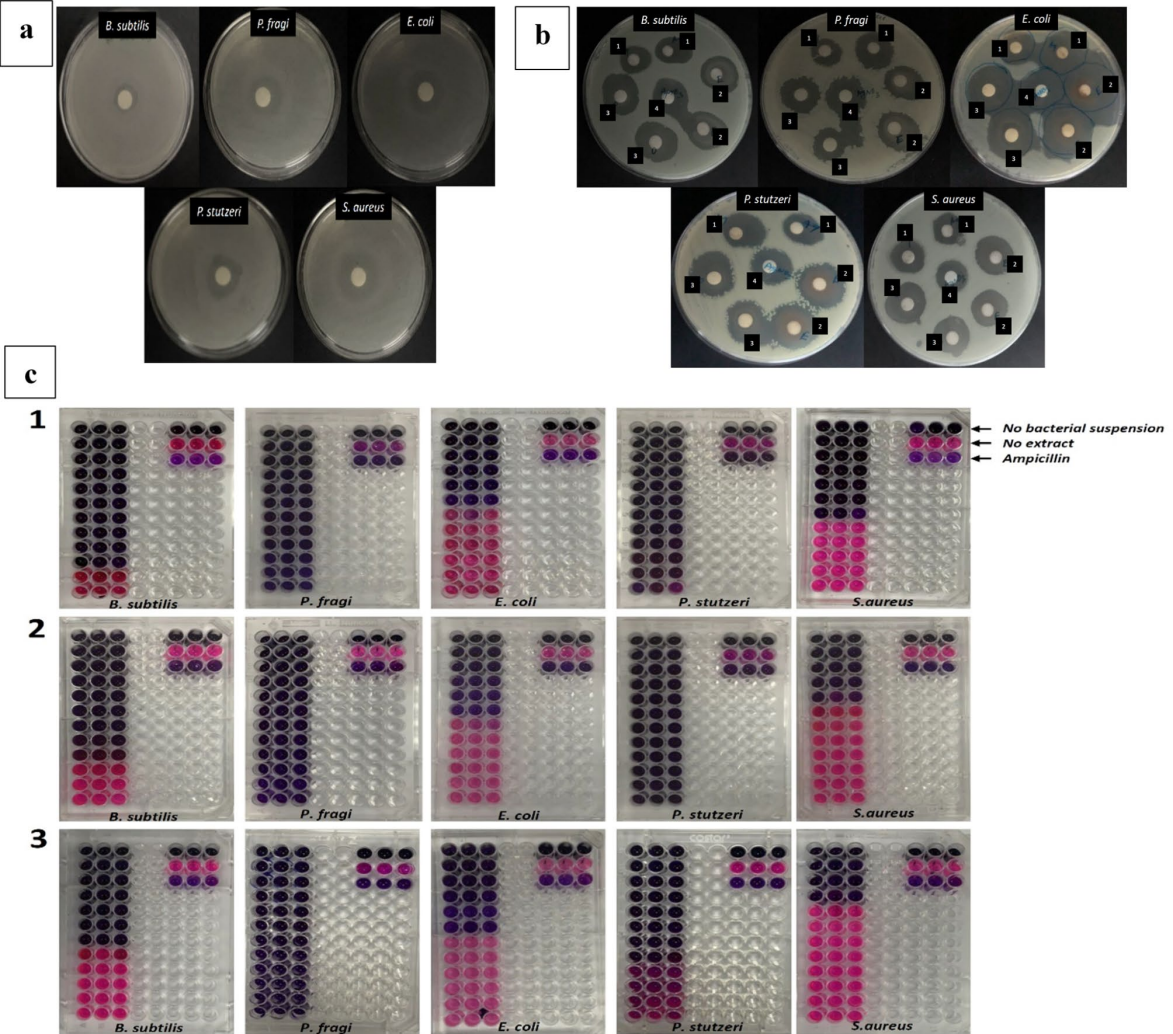
(**a**) Zone of inhibition of ampicillin on each bacterium, (**b**) Mueller Hinton agar plates with zone of inhibitions for the tested bacteria with (1) AQ-AgNP, (2) ET-AgNP, (3) MCF-AgNP, and (4) AgNO_3_ (**c**) MICs of the tested bacteria by (1) AQ-AgNP, (2) ET-AgNP, and (3) MCF-AgNP.

The biosynthesized AgNPs were tested against 5 bacterial strains (Table 2). AQ-AgNP showed highest inhibition for *P. fragi* (22.5 mm) followed by *E. coli* (22 mm), *P. stutzeri* (19.5 mm), *S. aureus* (18 mm), and *B. subtilis* (17 mm). A similar trend was seen for ET-AgNP with maximum inhibition of *P. fragi* (25 mm), *E. coli* (24.5 mm), *P. stutzeri* (23.25 mm), *B. subtilis* (20 mm), and *S. aureus* (19.25 mm). Lastly, MCF-AgNP recorded the maximum inhibition for *E. coli* (23.5 mm), *P. fragi* (21.75 mm), *P. stutzeri* (20.5 mm), *B. subtilis* (20.25), and *S. aureus* (19 mm) respectively. Figure 8 shows the zone of inhibition for each bacterium with positive control ampicillin (Fig. 8a) and inhibition zones for the three NPs and AgNO_3_ (Fig. 8b). The MICs of all the bacterial strains with the three biosynthesized NPs are shown in Fig. 8c.

**Fig. 9:**
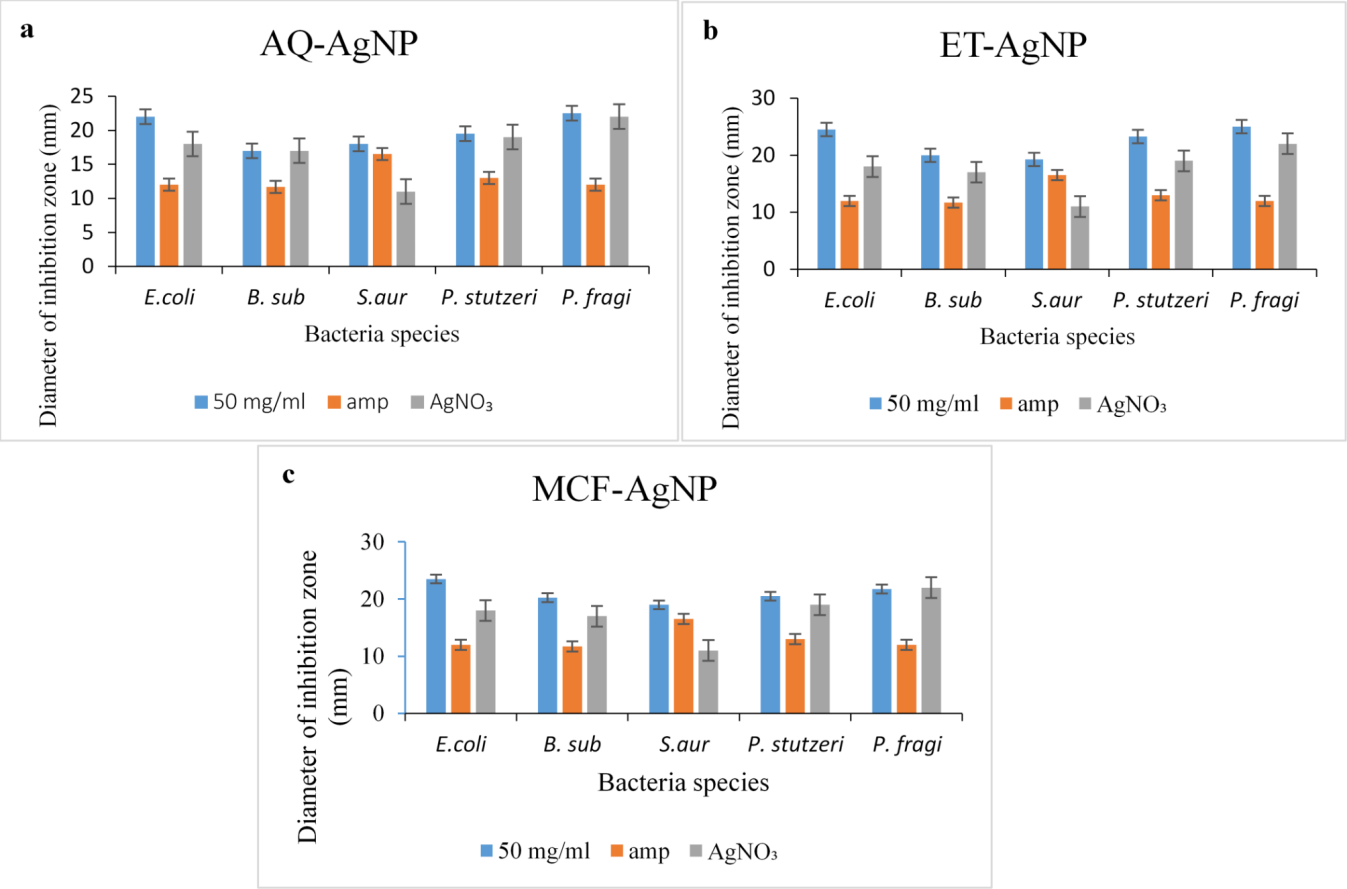
Diameter or inhibition zone (with standard error bars, *N* = 3) of (**a**) Aqueous Silver Nanoparticles, (**b**) Ethanolic Silver Nanoparticles, and (**c**) Methanol-Chloroform Silver Nanoparticle against 5 species by disc diffusion method.

Further, the MIC was performed for all bacterial strains with three AgNPs, the findings are compiled in Table 2. The values of inhibition with the standard error bars are shown in Fig. 9. The MIC for *E. coli* for the three types of AgNP’s is 3.13 mg/mL. For both *P. stutzeri* and *P. fragi*, the MIC recorded is 0.04 mg/mL. For *B. subtilis*, the MIC is 0.19 mg/mL, 6.25 mg/mL, and 12.5 mg/mL for AQ-AgNP, ET-AgNP, and MCF-AgNP. For *S. aureus*, the MIC is 1.56 mg/mL, 0.39 mg/mL, and 1.56 mg/mL for AQ-AgNP, ET-AgNP, and MCF-AgNP respectively. Ampicillin, being an antibiotic, showed inhibition zones while AgNO_3_ showed inhibitory zones much smaller than the NPs indicating towards the inhibitory nature of Ag^+^ ions. In summary, it can be concluded that antibacterial activity of ET-AgNP is higher than the MCF-AgNP which is higher than AQ-AgNP.

**Table 2:** (a) zone of inhibitions *±* SE of AgNPs obtained from aqueous, ethanolic, and methanol-chloroform extracts of macroalgae (b) minimum inhibitory concentrations (mg/mL) for all the three nanoparticles against different bacterial strains.

	*E. coli*	*B. subtilis*	*S. aureus*	*P*. *stutzeri*	*P*. *fragi*
a. Antibacterial Activity (mm)					
AQ-AgNP	22 ± 0.58	17 ± 0.58	18 ± 0.58	19.5 ± 0.58	22.5 ± 1.44
ET-AgNP	24.5 ± 0.29	20 ± 0	19.25 ± 0.43	23.25 ± 0.72	25 ± 0
MCF-AgNP	23.5 ± 0.87	20.25 ± 0.14	19 ± 0.29	20.5 ± 0.29	21.75 ± 0.43
Ampicillin	12 ± 0.29	11.7 ± 0.03	16.5 ± 0.29	13 ± 0	12 ± 0.29
AgNO_**3**_	18 ± 0.32	17 ± 0.73	11 ± 0.74	19 ± 0.32	22 ± 0.39
b. Minimum inhibitory concentration (mg/mL)					
AQ-AgNP	3.13	0.19	1.56	0.04	0.04
ET-AgNP	3.13	6.25	0.39	0.04	0.04
MCF-AgNP	3.13	12.5	1.56	0.78	0.04

### Mechanism of action of bacterial inhibition

**Fig. 10:**
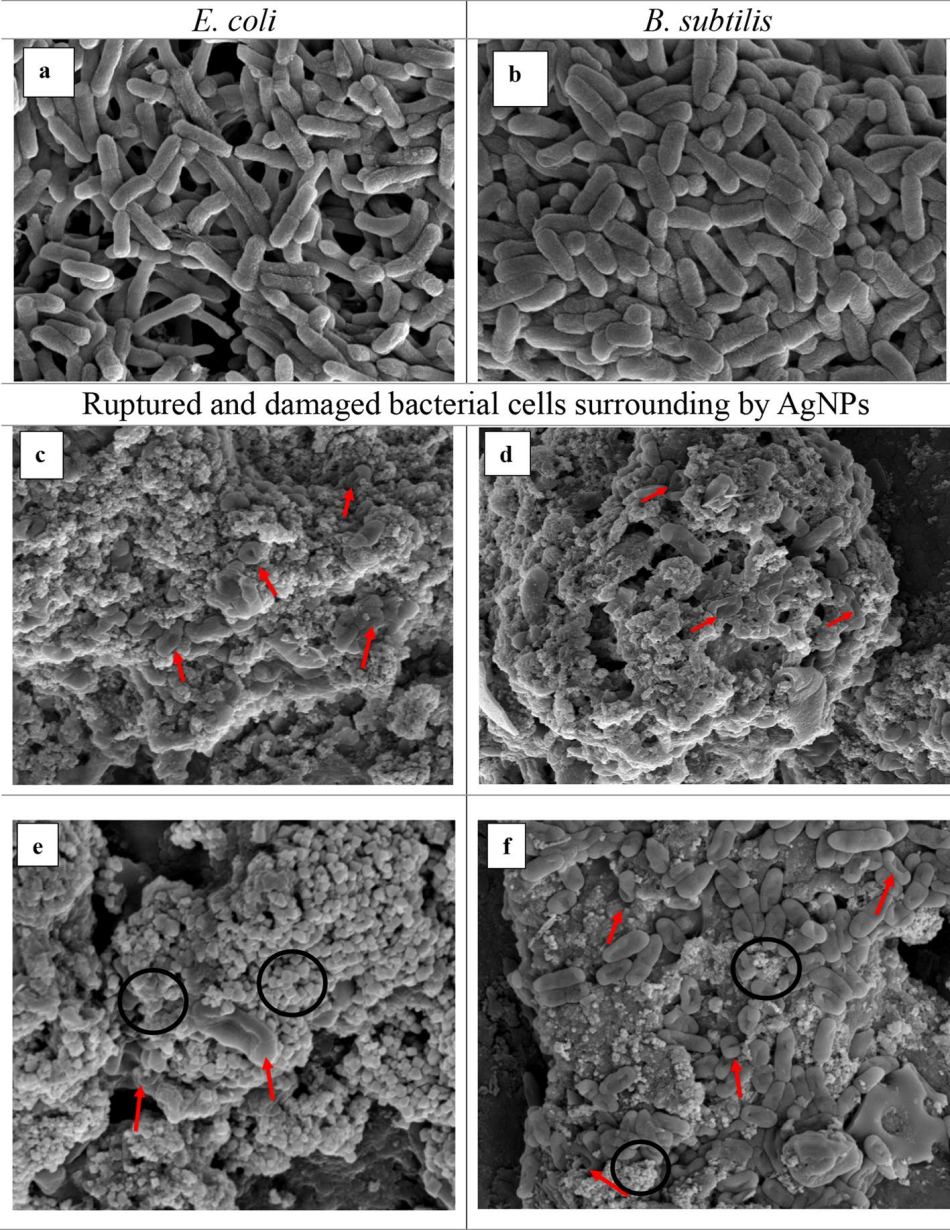
SEM images of bacterial cells without and with treatment of ET-AgNPs (Magnification: 25,000x). *E.* coli: non-treated (**a**) and treated (**c**, **e**). *B. subtilis*: non-treated (**b**) and treated (**d**, **f**).

To investigate how the AgNPs react once in contact with the bacterial cells, SEM analysis was carried out. The SEM images showed clear differences between the treated (Fig. 10c-f) and untreated cells (Fig. 10a & b). Both *E. coli* and *B. subtilis* showed deformed and ruptured cells compared with controls that appeared to be smooth, integrated, and intact. At some regions in the SEM images, there are gaps and pits on the cells’ surface (marked with red arrows in Fig. 10c & d). The deformed and damaged cells are indicated by red arrows while AgNPs surrounding the bacterial cells are also found in SEM images circled black (Fig. 10e & f).

## Discussion

Macroalgae is home to many biological compounds with the utmost benefits for mankind. It is imperative to analyze the different biologically active molecules and secondary metabolites that exist in seaweeds. For this purpose, in this research, three different types of solvents were used to extract bioactive compounds that were utilized to biologically synthesize NPs. Water as a solvent is the most polar which is mainly used to extract polar compounds, followed by ethanol which is used to extract compounds like tannins, polyphenols, alkaloids, flavanol, and terpenoids^65^. Lastly, the chloroform and methanol solvents together are used to extract lipids. In this mixed solvent, methanol acts as a disrupter breaking the strong bonds between lipids and proteins while chloroform acts as a mediator that initiates the diffusion processes and extraction of lipids from the biomass^66^. The extracts from macroalgae were used to synthesize NP’s that indicate the involvement of the extracted compounds in stabilizing and capping of NPs.

UV-VIS spectroscopy is a technique used as a preliminary confirmation of AgNPs synthesis. This technique is not only reliable but also easy and efficient for different metal synthesized NPs^67^. In the present study, the absorption peaks for AQ, ET, and MCF-NPs obtained are 422 nm, 423 nm, and 423 nm respectively. Our study aligns with other available research where the absorbance peaks of majority of the biosynthesized AgNPs are between 420 nm and 450 nm^68,69^. Similarly, in another study seaweeds *S. polycystum*,* Acanthophora spicifera*, and *S. wightii* were used to biosynthesize AgNP’s, when analyzed with UV-VIS, the peaks appeared at 424 nm, 409 nm, and 415 nm consecutively^70^. Furthermore, fresh and dry seaweed *Codium capitatum* was used to synthesize AgNPs, the UV-VIS peaks appeared at 422 nm and 425 nm^71^. *S. cinereum* was also used to synthesize AgNP and its absorption peak in UV-VIS was seen at 408 nm^72^. In another study, *Moringa oleifera* leaves were used to synthesize AgNPs with absorbance peak at 419 nm in UV-VIS spectrum^2^.

The stability, the efficiency of the reduction process of Ag^+^ during the biosynthesis of NPs, and its antimicrobial activities also depend on its working pH. In our study, the working pH of AQ, ET, and MCF-AgNP were 6.5, 7.29, and 6.22. Generally, NPs synthesized at low pH are unstable and exhibit weaker antimicrobial activities as compared with NPs synthesized at neutral and higher pH’s^73^. Apart from the stability, the morphology of the biosynthesized NPs is also affected by the pH of the system. For instance, a study reports the sizes of biosynthesized AgNPs to be 80 nm, less than 50 nm, and less than 35 nm at pH’s of 7, 9, and 11^74^. Other studies report that the acidic and neutral working pH’s help in synthesizing NPs with greater sizes and aggregation. However, some studies consider pH 7 and pH 8 to be optimum to produce AgNPs^75,76^. Generally, basic or alkaline conditions provide more functional group charges, thus, accelerating the reduction of Ag^+^ ions.

The TEM analysis showed that the biologically synthesized AgNPs have spherical shape. TEM analysis is commonly used to evaluate the morphology and size of the synthesized NPs^77^. Previously, our team chemically synthesized AgNPs using aqueous extract which also confirmed spherical shape of the NPs, however, the size varied between 5 nm and 50 nm^42^. A comparable study involving the aqueous extract of *Portieria hornemannii* to prepare NPs reported its size to be in range of 60–70 nm^78^. Other study where ethanolic extract of *G. birdie* was used to prepare NPs, also had sizes ranged between 20.2 and 94.9 nm^79^.

The functional groups present in the synthesized NPs were evaluated using FTIR analysis. FTIR analysis, nowadays, is used widely to obtain the functional groups present within the biological compounds^80^. The functional groups shown in the FTIR spectra correspond to the extracted compounds with possible interaction with AgNO_3_ to synthesize NPs and then initiate the capping and stabilizing processes. The capping agents are mainly responsible for the stability of biosynthesized NPs by controlling several factors such as their size, agglomeration, and shape. Typically, the surface energy of NPs is high leading to surface aggregation of the particles^81^. Efficient capping agents play a role in reducing the surface energy of NPs, hence, preventing agglomeration. In our results, the NPs synthesized from ethanolic extract showed less agglomeration as compared to aqueous and methanol: chloroform extracts making it most efficient compared to the other two extracts. In our study, the peaks in the FTIR spectra of the extracts (Fig. 3) indicates the presence of different functional groups that assists in reduction of Ag^+^ to Ag^0^ and in the capping of the biosynthesized AgNPs^34,82^. In AQ-AgNP, the broadband for O-H group has disappeared when compared with its control AQ extract. This means the compounds with hydroxyl functional group are involved in the reduction of Ag^+^. From the spectra, it can also be reported that the AQ-AgNP has no new or intense peaks which could be one of the reasons for the NPs to have less efficient capping and poor stability leading to agglomeration. This can also be confirmed and supported through the TEM analysis image (Fig. 2b) where the NPs are seen to be agglomerated. Spectra of ET-AgNP and MCF-AgNP shows intense broad bands for hydroxyl functional groups as compared to their controls denoting the involvement of this group in stabilization and capping processes of AgNPs. Similarly, functional groups labeled 1 and 3 in the FTIR spectra of ET-AgNP and MCF-AgNP intensified as compared to the controls showing that they play role in capping process and post-synthesis stability of AgNPs. Peak labelled 2, however, appeared to be slightly varied in the NPs spectra. MCF-AgNP FTIR spectrum shows a peak for hydroxyl stretch while other peaks belong to non-alcoholic functional groups indicating the presence of lipids which was expected due to the solvent (methanol: chloroform) used for extraction. The functional groups found in FTIR spectrum relate to the compounds present within the macroalgae. For instance, the presence of hydroxyl, amide, and ester groups eventually represent the existence of phenols, proteins and/or amino acids, and lipids^83^ in the seaweeds.

XRD analysis is a method used to gather information about the sample, including its purity, crystal size, and lattice distortion^84^. Generally, the XRD spectra reveals the intensity of scattered X-rays from the samples at 2θ positions. The intensity of the peaks indicates the atom number involved in scattering X-rays and the crystallinity extent of the sample. Crystallinity extent indicates the higher degree of crystallinity of the sample that is represented by a peak with greater intensity in the XRD pattern^85^. These crystallographic planes are characteristics of face-centered cubic (fcc) structure of metallic Ag. The samples show intense (111), and (002) reflections indicating that most NPs have a (111) and (002) plane^86^ (Fig. 4). All these well-defined and strong peaks confirmed that the AgNPs formed have crystalline structure based on the XRD spectra.

In this study, the NPs were characterized with various techniques. One of the techniques used is GC/MS. The GC/MS spectrums obtained for the biosynthesized NPs indicate the presence of numerous beneficial compounds. Several compounds identified in the GC/MS analysis are also reported in the literature such as Hexadecenoic acid or 2-hydroxy-1-(hydroxymethyl) ethyl ester. This compound has been reported previously in the GC/MS analysis of brown macroalgae *Hydroclathrus clathratus*^87^. Compound 9-Octadecynoic acid, methyl ester has been identified previously in the methanolic extract of *U. fasciata*^88^. Other compounds such as Aromandendrene, known for its antibacterial activity^89^ and hexadecenoic acid and its derivatives known to exhibit additional biological activities, including antioxidant and anti-inflammatory properties^90^ are also found in macroalgae. These compounds are found in the GC/MS spectra of our studied macroalgae and indicate their role in antibacterial activity of NPs. Seaweed is rich with compounds that are nutritionally significant as well as holding pharmaceutical importance. Polysterols are among the important chemical compounds that are found to be present in macroalgae. Polysterols are found in brown macroalgae with a small proportion of cholesterol^91^. Our GC/MS results confirmed the presence of one of the polysterols; campesterol in both ET-AgNP and MCF-AgNP spectra. Similarly, in the MCF-AgNP spectrum, peak for cholesterol has also been detected and identified. In literature, several species of seaweeds are reported to have campesterol and cholesterol such as *Saccharina latissimi*^92^, *Ecklonia radiata*^93^, *Padina australis*^94^, *Porphyra dentata*^95^, and *S. piluliferum*^96^. Polysterols are significant role players in biomedicine due to its unique antibacterial properties. For instance, cholesterol derivative compound extracted from *Laurencia papillosa*, a red macroalgae is known for its inhibitory action against *S. aureus*,* E. coli*,* P. aeruginosa*,* K. pneumoniae*, and *Shigella flexineri*^97^. Similarly, green algae, *C. cylindacea* has been found to have higher proportion of polysterols such as campesterol, stigmasterol and so on. These compounds are evaluated for their antibacterial properties and found to posses’ notable antibacterial activities^98^. Likewise, red and green macroalgae, brown macroalgae such as *H. cuneiformis*,* Turbinaria* spp, and *Sargassum* spp. L are also reported for their antibacterial properties. The extracts of these macroalgae revealed the presence of many polysterol compounds including the derivatives of cholesterol^99^. Furthermore, the presence of the listed compounds in the NPs as seen in the GC/MS spectra, indicates their involvement in the neutralization and stabilization process of AgNPs. Initially, the functional groups present in the compounds reduces and then stabilizes the NPs^100^. Generally, the biosynthesis of NPs occurs in three major stages: (1) the activation stage, (2) the growth stage, and (3) the termination stage. In the first step, the metal ion undergoes reduction, followed by its nucleation and the growth stage where small NPs group together to form larger NPs in line with thermodynamic stabilization. In the last stage; the termination stage, the final shape of NP is defined^101^. Further, these compounds contribute to the synthesis of NPs by adsorbing them on their surface. This adsorption process on the surface of NPs lead to substitution with functional groups (–OH, –NH_2_, –COOH, –NO_2_) from the active metabolites^102^.

To evaluate further the composition of the biosynthesized AgNPs, XPS analysis was performed. XPS is a tool primarily used to investigate the chemical nature and composition of the nanomaterials^103^. By knowing the chemical nature (state of oxidation) of the elements present in the NP, its interaction with the surrounding compounds or environment can be evaluated.

The antibacterial activity of AgNPs clearly demonstrates their inhibitory effect on bacteria. The precursor AgNO_3_ (2mM) that was used to synthesize NPs showed clear and smaller zone of inhibitions indicating the inhibitory nature of Ag^+ ^[10] The shapes and sizes of the synthesized AgNPs are dependent on the concentration of AgNO_3_ used. It is reported that NPs synthesized with 5 mM AgNO_3_ have higher inhibitory effects than NPs synthesized with 2 mM AgNO_3_. AgNPs had inhibitory effects against Gram-positive such as *S. aureus*, and Gram-negative bacteria like *E. coli* which aligns with our research. AgNPs synthesized from aqueous extract of brown macroalgae, *S. muticum* showed inhibitory activities against *B. subtilis*,* K. pneumoniae*, and *S. typhi* through agar disc diffusion method. It was observed that the zone of inhibition expanded as the concentration of AgNPs^104^ increased. Aqueous extract of red algae, *Gelidium amansii* was used to synthesize AgNPs and were tested against *S. aureus*,* B. pumilus*,* E. coli*,* P. aeruginosa*,* Vibrio parahaemolyticus*, and *Aeromonas hydrophila*^105^. Similarly, another species of genus Gelidium, *G. corneum* is also used for synthesis of AgNPs that inhibited the growth of *E. coli*^106^. Ethanolic extract of red algae *A. specifera* was used to prepare AgNPs, the efficacy of which was tested against *S. aureus*, *B. subtilis*,* Salmonella* spp., and *E. coli.* The synthesized NPs inhibited the growth of all the tested species with maximum inhibition zone for *E. coli*^107^. In a different study, AgNPs were synthesized from chloroform: methanol (1:1 v/v) extract of brown macroalgae; *Spatoglossum asperum* and *Hedophyllum sessile*. Both AgNPs were tested and proved efficient against pathogenic bacteria; *Xanthomonas axonopodis* pv. *Citri* and *X. oryzae* pv. *oryzae*^108^.

The mechanism through which AgNPs inhibit the growth of bacteria is controversial. It has been proposed that AgNPs either inhibit bacterial growth or kill the bacteria by harming the membrane, causing DNA damage, generating reactive oxygen species (ROS), denaturing proteins, and inactivating enzymes^71,109^. The SEM micrographs indicates that AgNPs induced changes in the membrane of the bacterial cells effecting the transport mechanisms of bacteria leading to cell death. Some studies suggest that the AgNPs initiate bacterial cell destruction through damaging the cell membrane and then entering the inner membrane followed by the production of ROS, thus, inhibiting the cells growth^110,111^. Apart from generation of ROS, it is reported that inactivity of cells could also be related to the denaturation of proteins that happens when Ag^+^ from AgNPs bind to sulfhydryl groups of the proteins^112^. Furthermore, the NPs, once in contact with bacterial cells, start to accumulate on their membrane leading to structural or morphological alterations. Such alterations include cytoplasm shrinkage, membrane detachment, and cavities or pits^113^. There are different speculations about the effect of AgNPs on both Gram-positive and Gram-negative bacteria. The presence of thick layer of peptidoglycan in Gram-positive bacteria lessen the influence of AgNPs whereas Gram-negative bacteria are affected adversely^114^. On the other hand, some studies suggest the opposite. In a study conducted by Premanathan and his colleagues^115^, Gram-positive bacteria *S. aureus* were affected by NPs more than the Gram-negative bacteria *E. coli.* Our results align with the previously mentioned result as *B. subtilis* had more intense cavities and pits as compared with *E. coli.*

### Method

### Chemicals

Formalin (Riedel-de Haën), Ethanol (Merck), Methanol (Merck), Chloroform (LOBA CHEMIE PVT. LTD), Silver nitrate (Scharlau), Resazurin (Signa-Aldrich), Nutrient Agar (Condalab), Nutrient Broth (HiMedia), Mueller Hinton Agar (Condalab), Glutaraldehyde (VWR), Phosphate Buffer Saline (Sigma-Aldrich).

### Sample collection

*Hormophysa triquetra* (HT) was collected from Ad-Dukhan (25°30’10.8”N 50°50’05.4"E), Qatar in June 2023. The species were picked manually, rinsed with seawater to remove other macroalgae and debris and were relocated to lab in thermocol box.

### Sample preparation

The macroalgae was washed with Milli-Q water thrice upon transferring to the laboratory and then shade dried^116^. After drying, the samples were grinded to obtain powder. The powder was refrigerated at 4 °C until further use. The sample of macroalgae was preserved in 4% Formalin and in dried form in the Department of Biological and Environmental Sciences, Qatar University, Doha, Qatar.

### Biosynthesis of AgNPs

To biosynthesize AgNPs, AQ, ET and MCF extracts of the macroalgae were prepared as described in the sections below:

### Preparation of AQ extract

To prepare AQ extract of macroalgae, 30 g of dried macroalgal powder was added to 300 mL of sterile distilled water^10^ and was incubated in water bath at 60 °C for 20 min with constant shaking^117^. The extract was then filtered by Whatman filter paper to be used for the biosynthesis of AgNPs.

### Preparation of ET extract

To prepare ET extract of macroalgae, 30 g of macroalgal powder was added to 300 mL of 70% ethanol. The mixture was kept at room temperature in a shaker at 120 rpm for 24 h. Upon completion of incubation, the extract was filtered, and another 100 mL of 70% ethanol was added to the macroalgal biomass until no further color change was observed^118^. The solution was filtered, and the filtrate was oven-dried at 40 °C in sterile glass plates. The extract powder was stored at **−** 22 °C until further use.

### Preparation of MCF extract

To prepare MCF extract of macroalgae, 30 g of powder was added to 300 mL of a mixture of methanol and chloroform in a ratio of 1:2^119^. After the completion of sequential extraction, the extracts were oven-dried at 40 °C in sterile glass plates. The extract powder was stored at **−** 22 °C until further use.

### Biosynthesis of AgNPs

AgNPs were prepared from all the three types of extract powders based on the procedures described in this section. For the preparation of AgNPs, 2 mM (0.34 g/L) of AgNO_3_ stock solution was prepared. To 20 mL of fresh AQ extract, 180 mL of 2 mM AgNO_3_^10^ was added and mixed in a flask. The flask was covered with foil and was incubated at 85 °C for 24 h^68^. Color changes were observed at the end of incubation, and the pH was measured following which the mixtures were centrifuged for 1 h at 5000 rpm. The synthesized NPs were washed with sterile distilled water thrice to remove any residual or unbound particles^70^. The resultant NPs were oven-dried at 50 °C.

For the biosynthesis of AgNP from ET and MCF extract powders, 200 mg of the powders were added to 100 mL of AgNO_3_ solution each. The mixtures were stirred for 2 min. After the change of color, the mixtures were centrifuged for 30 min at 5000 rpm followed by washing with sterile distilled water thrice^120^. The NPs synthesized were oven-dried at 50 °C.

### Characterization of AgNPs

To confirm the formation of AgNPs, the absorbance of the biosynthesized AgNPs were measured between the range of 200 nm to 800 nm using UV-VIS spectrometer (PerkinElmer Lambda 25 UV/VIS). AgNO_3_ was used as blank. TEM (Tecnai G2 TEM, TF20, FEI, Oregon, USA) was used to analyze the NPs’ structural properties. FTIR (Spectrum 400 Perkin Elmer) between the range of 400 cm^− 1^ to 4000 cm^− 1^ was used to investigate the functional groups of the NPs. Empyrean XRD device was utilized to carry out the XRD analysis that can scan the samples between the range of 5° ≤ 2θ ≤ 90°. The anode material used in the device is Cu that has a radiation of 1.5425 Å. The crystallite grain size (D) was calculated based on Debye-Sherrer Equation^121^:

$$\:\text{D}\:=\frac{k\lambda\:}{\beta\:cos\theta\:}$$

$$\beta\:=full\:width\:at\:half\:maxima\:\left(FWHM\right)$$

$$k=Sherrer\:constant\:$$

$$\theta\:=Bragg\:diffraction\:angle$$

$$\lambda\:=wavelength\:of\:x-ray$$

GC/MS analysis was carried out to find the compounds present in NPs. For analysis, GC system 7890 A coupled with 5973 network mass-selective detector (Agilent, Santa Clara, CA, USA) in scan mode with mass range between 45 and 500 m/z was used. Helium was used as a carrier gas with a flow rate of 1.50 mL/min. For sample preparation, 0.5 g of each NP was added to 1 mL of dichloromethane in an eppendorf tube. The tube was heated at 35 °C to obtain a suspension. The suspension was further diluted by adding 10 mL of dichloromethane. After mixing well, it was filtered with 0.2 μm syringe filter. About 1 µL of sample was injected into the Rxi-5Sil MS GC column (30 m, 0.32 mm ID, 0.25 μm) in a split ratio of 10:1. The column temperature was set at 80 °C and then increased to 250 °C at a rate of 15 °C per mins. The compounds were identified based on comparison with NIST 20 library.

### Antibacterial activities of biosynthesized AgNPs

The antibacterial activities of AQ-AgNP, ET-AgNP, and MCF-AgNP were tested against *E. coli*, *B. subtilis*, *S. aureus*, *P. fragi*, and *P. stutzeri*. The bacteria were cultured on nutrient agar for 24 h. A microbial lawn of each bacterium was spread on Mueller-Hinton agar^118^. Sterile susceptibility discs were inoculated with 30 µL of AgNPs (50 mg/mL) and AgNO_3_ (2mM). Ampicillin (200 µg) was used as positive control for the experiment.

### Determination of minimum inhibition concentration

The MIC of all the three NPs against five bacterial strains were determined using 96 well plate method^118^. Each well was inoculated with 100 uL of nutrient broth. Different concentrations of AgNP from 0.04 mg/mL to 100 mg/mL were evaluated. Wells were inoculated with bacterial suspensions and all the species were tested in triplicates. Clear media and ampicillin were used as negative and positive controls. Indicator dye resazurin (stock: 0.27 g/40 mL distilled water) was loaded in all the wells and was used to monitor the growth of bacteria. Resazurin is non-fluorescent blue dye which converts into pink-fluorescent dye upon detecting active cells^122^. MIC is defined as the lowest concentration where resazurin dye does not change color during incubation^123^. The wells were incubated at 37 °C for 24 h after which the results were recorded.

#### Mode of action of bacterial inhibition

To study the mode of action of bacterial inhibition by AgNPs, ET-AgNPs were used against *E. coli* and *B. subtilis.* Suspensions of both the bacterial strains along with the NPs with concentrations double the MIC values (Table 2b) were added to nutrient broth and then incubated at 37 °C in a shaking incubator at 198 rpm for 1 h. Table 3 shows the total volumes of suspension and nutrient broth added along with AgNPs.

**Table 3:** Details of the quantities of suspension, NPs, and nutrient broth used to study the mode of action of NPs against *E. Coli* and *B. subtilis.*

	Suspension (µL)	NP’s (2 MIC) (µL)	Nutrient Broth (µL)
Control *E.coli*	300	0	1700
*E. coli* + AgNP	300	200 (6.26 mg/mL)	1500
Control *B. subtilis*	300	0	1700
*B. subtilis* + AgNP	300	200 (12.5 mg/mL)	1500

After incubation, the cultures were centrifuged at 6000 rpm for 10 min. For fixation steps, methodology of Amdadul Huq^124^ was followed with slight modification (only used 1 fixative-glutaraldehyde without using Osmium tetroxide). The pellet obtained was washed with phosphate buffer saline (PBS) thrice. The pellet was fixed with 2.5% glutaraldehyde for 2 h. The sample was washed again with PBS three times followed by graded dehydration with increasing ethanol concentrations for 10 min each (25%, 50%, 70%, 80%, 90%, and 100%). The samples were then smeared on metallic studs and then gold coated to be observed under Nova NanoSEM 450.

## Conclusion

In conclusion, the macroalgal extracts can be effectively used to biologically synthesize AgNPs, which were visually confirmed by noticeable color changes and further validated by UV-VIS spectroscopy. Characterization of the AgNPs revealed a spherical shape with a size ranging between 10 nm and 70 nm. Additional studies indicated the presence of various chemical compounds with functional groups such as hydroxyl, aliphatic, aromatic, and ether groups, which play a role in the capping process of silver. The AgNPs synthesized from all extracts demonstrated antibacterial activity against the tested bacterial species, with inhibition zones ranging from 17 mm to 25 mm. The mechanism of the antibacterial activity was demonstrated by SEM analysis which showed that the NPs inhibited the growth of the bacteria by damaging the cell’s morphology. This study suggests that macroalgae could be a promising candidate for the sustainable production of AgNPs with antibacterial properties. This study will act as a baseline for any future macroalgal-based studies in Qatar for it being an eco-friendly approach for synthesis of NPs.

## Data Availability

The datasets used and/or analysed during the current study are available from the corresponding author on reasonable request.
